# Detection of left ventricular regional dysfunction using strain analysis by feature tracking of Cine MRI in rheumatoid arthritis patients without cardiac symptoms

**DOI:** 10.1186/1532-429X-14-S1-P191

**Published:** 2012-02-01

**Authors:** Yasuyuki Kobayashi, Yokoe Isamu, Masaharu Hirano, Hitomi Kobayashi

**Affiliations:** 1 Radiology, St.Marianna University School of Medicine, Kawasaki, Japan; 2 Rheumatology, Itabashi Chuo General hospital, Tokyo, Japan; 3 Cardiology, Tokyo Medical Colleage, Tokyo, Japan

## Background

In patients with rheumatoid arthritis (RA), cardiac involvement is common. This cardiac involvement may have serious consequences and can contribute to worsening of patient’s outcome. Cardiac MRI is a sensitive, non-invasive diagnostic techniques that can identify subclinical myocardial abnormalities. We sought to detect LV regional dysfunction by using a CMR approach in RA patients without cardiac symptoms.

## Methods

Consecutive patients with RA and healthy control subjects were enrolled. RA patients received either non-biologic DMARDs or biologics. All subjects without cardiac symptoms underwent non-contrast CMR on a 1.5 T scanner(Philips). Peak systolic regional radial strain (Err, %) was calculated by feature tracking of cine MRI(ZIOsoft, CA, USA). We explored the associations of peak Err between the control and RA groups. Comparisons of peak Err between the DMARDs and the biologics groups, and the association of peak Err with disease activity and severity measures were determined. We compared 28 RA patients (mean age 58.0±12.6 years) with 10 non-RA controls (mean age 55.7±4.6 years). RA patients received either DMARDs or infliximab or tocilizumab.

## Results

Mean peak Err of all segments was lower in RA patients than in normal subjects but not significantly different (p=0.07). Peak Err in both the anterolateral and inferior wall was significantly lower in RA patients than in controls (p=0.01, p=0.01, respectively). In the DMARDs group, mean peak Err was significantly lower than in the biologics group (p=0.02). Mean peak Err was higher in the TCZ group than in the DMARDs group (p=0.01). Abnormal peak Err in RA patients was associated with higher mHAQ scores (R2=0.31).

## Conclusions

Our findings suggested sub-clinical LV regional dysfunction in RA patients without cardiac symptoms. Higher mHAQ scores might be an independent risk factor for myocardial involvement in RA. Our results indicated that TCZ might affect the normalization of LV regional function compared with non-biologic DMARDs.

## Funding

No disclosure.

